# Epidemiology, host range, and associated risk factors of monkeypox: an emerging global public health threat

**DOI:** 10.3389/fmicb.2023.1160984

**Published:** 2023-05-05

**Authors:** Munib Ullah, Yanmin Li, Kainat Munib, Zhidong Zhang

**Affiliations:** ^1^State Key Laboratory of Veterinary Etiological Biology, Lanzhou Veterinary Research Institute, Chinese Academy of Agricultural Sciences, Lanzhou, China; ^2^Department of Clinical Studies, Faculty of Veterinary and Animal Sciences, PMAS-Arid Agriculture University Rawalpindi, Rawalpindi, Pakistan; ^3^College of Animal Husbandry and Veterinary Medicine, Southwest Minzu University, Chengdu, China; ^4^Department of Sociology, Allama Iqbal Open University Islamabad, Islamabad, Pakistan

**Keywords:** epidemiology, monkeypox, host range, risk factors, global health, threat

## Abstract

Based on recent multiregional epidemiological investigations of Monkeypox (MPX), on 24 July 2022, the World Health Organization declared it a global public health threat. Retrospectively MPX was an ignored zoonotic endemic infection to tropical rainforest regions of Western and Central African rural communities until a worldwide epidemic in May 2022 verified the potential threat of monkeypox virus (MPXV) to be propagated across the contemporary world via transnational tourism and animal movements. During 2018–2022, different cases of MPX diagnosed in Nigerian travelers have been documented in Israel, the United Kingdom, Singapore, and the United States. More recently, on 27 September 2022, 66,000 MPX cases have been confirmed in more than 100 non-endemic countries, with fluctuating epidemiological footprinting from retrospective epidemics. Particular disease-associated risk factors fluctuate among different epidemics. The unpredicted appearance of MPX in non-endemic regions suggests some invisible transmission dynamic. Hence, broad-minded and vigilant epidemiological attention to the current MPX epidemic is mandatory. Therefore, this review was compiled to highlight the epidemiological dynamic, global host ranges, and associated risk factors of MPX, concentrating on its epidemic potential and global public health threat.

## 1. Introduction

The re-emergence of various transmissible infections, including Zika virus, swine flu (H1N1), Ebola virus, Nipah virus, avian influenza (H5N1), severe acute respiratory syndrome coronavirus 2 (SARS-CoV-2), Middle East respiratory syndrome coronaviruses (MERS-CoV), and recent regional outbreaks of monkeypox virus (MPXV) in the twentyfirst century, is alarming (Mourya et al., 2019). This spillover of viruses from animal origin to humans has predominantly been due to species barrier crossing (Bezerra-Santos et al., 2021). At a time when global health experts and world communities were awaiting the pandemic spread of coronavirus disease 2019 (COVID-19) to be diminished, contemporary global populations now face an unexpected MPX epidemic. In previous decades, irregular outbreaks, with thousands of MPX cases, have been predominantly limited to African countries. MPX is enzootic in several sub-Saharan states and has co-occurred in African inhabitants for several years but has not received sufficient consideration from global technical experts. Excitingly, MPX, for the first time, acquired worldwide consideration when it appeared in the USA in 2003 (Reed et al., 2004). Such sporadic and confined occurrences of MPX in the non-enzootic world have been associated with international travel and the importation of infected animals (Reed et al., 2004). Although MPXV spread among humans has been soundly investigated, its extensive concurrent appearance in non-enzootic nations has hit the globe with another shock. Additionally, MPX epidemics have been poorly inspected, irregularly reported, and poorly epidemiologically defined in the past, and, ultimately, the picture of this infection is incomplete. This menace can intensify with temporal patterns in the case where there is a rise in virulence naturally or through genomic rearrangement, a spillover into extra extensively dispersed taxa, or entrance and cluster epizootics in non-enzootic states (Sklenovská and Van Ranst, 2018). All these risks are further worsened by enhanced desforestation, increasing population density, large-scale international travel, immigration, invasion and damage of natural animal habitations, and poor epidemiological approaches toward emerging and re-emerging disease investigations (Adler et al., 2022). Lately, MPX is making headlines due to the worldwide surge in the occurrence of the infection in many countries and continents. On 24 July 2022, the World Health Organization (WHO) declared MPX a global public health threat. As per the US Centers for Disease Control and Prevention report of 2 August 2022, ~25,391 clinically confirmed MPX cases have been documented across 87 states globally (CDC, 2022b). This number has been ballooning prospectively in the USA, Brazil, Spain, the UK, and Germany (WHO, 2022d). More recently, on 27 September 2022, over 66,000 confirmed MPX cases have been documented in more than 100 non-endemic states, with fluctuating epidemiological footprinting from retrospective epidemics. In this scenario, when the global occurrence of MPX does not decline, the world might see another pandemic, which may be presently hanging over the head of the contemporary world and may easily become a global public health threat. Therefore, this review assembles updated literature on the different aspects of MPXV regarding disease epidemiology, host range, and associated risk factor, and also sheds light on its epizootic potential and global public health threat. Restoring public health setups and preparing for upcoming epidemics are required, particularly in underdeveloped countries with deprived healthcare delivery services.

## 2. MPX

Monkeypox (MPX) is a sporadic zoonotic viral infection caused by the MPXV, which belongs to the genus *Orthopoxvirus* of the family Poxviridae and is interrelated to the already eradicated smallpox virus. It is a large, enveloped virus comprising a dsDNA genome of 190 kbp and having a dumbbell-shaped core with horizontal figures (Kugelman et al., 2014). The MPXV has two distinct genomic groups, the West African clade and the Congo Basin clade. These genomic groups have been geologically isolated with diverse clinical and epizootological characteristics (WHO, 2022d). The Congo Basin clade is recognized to induce serious infection and can spread among humans with a fatality rate of ~11%. However, the West African clade displays a fatality rate of <1% and has never been known to exhibit human-to-human spread (Jezek et al., 1987). The early signs and symptoms of MPX are frequent pyrexia, vigorous headache, myalgia, lymphadenopathy, and lethargy. After fever, the dermal wounds characteristically burst within 1 to 3 days. The rash tends to be more confined to the facial region and extremities as compared with the trunk region of the body. MPX is frequently a self-determining disease, and symptoms last from 2 to 4 weeks. The clinical appearance and indications of MPX are exactly like those of smallpox; however, it is a mild and rarely fatal infection (Soheili and Nasseri, 2022).

Monkeypox virus (MPXV) continues to present challenges to public health and healthcare providers in areas with endemic disease, owing to inadequate capacity to diagnose and clinically manage patients and to accurately identify exposures (McCollum, 2023). Mostly, MPX cases in the African subcontinent are mainly misdiagnosed with other zoonotic infections such as cutaneous anthrax, chickenpox (Varicella), staphylococcal-associated rash, or fungal diseases in cases with human immunodeficiency virus (HIV) infection (Formenty et al., 2010). In addition to the current outbreak, there have been multiple reports of initial misdiagnosis of patients who were later confirmed to have MPX (Heskin et al., 2022; Minhaj et al., 2022) due to an atypical clinical manifestation that does not resemble the MPX observed in African outbreaks. Laboratory evaluations for monkeypox cases include electron microscopy, immunohistochemistry, culture of material from rash specimens, serological testing for specific antibodies, and real-time or conventional polymerase chain reaction (PCR) assays. Confirmation of specimens from suspected MPX cases is performed using nucleic acid amplification testing, such as real-time or conventional polymerase chain reaction. Restriction fragment length polymorphism (RFLP) of PCR-amplified genes or gene fragments is also used to detect monkeypox DNA. However, this method is time-consuming and requires a virus culture. Whole-genome sequencing, using next-generation sequencing technologies, is the gold standard for the characterization of MPXV and other orthopoxviruses. However, the use of most of the above diagnostic tools is limited due to their high cost and advanced technology, especially in developing countries and regions with limited healthcare resources (MacNeil et al., 2011; Radonić et al., 2014; Brown and Leggat, 2016; Petersen et al., 2019b; Alakunle et al., 2020; Cohen-Gihon et al., 2020; Altindis et al., 2022).

**Figure 1:**
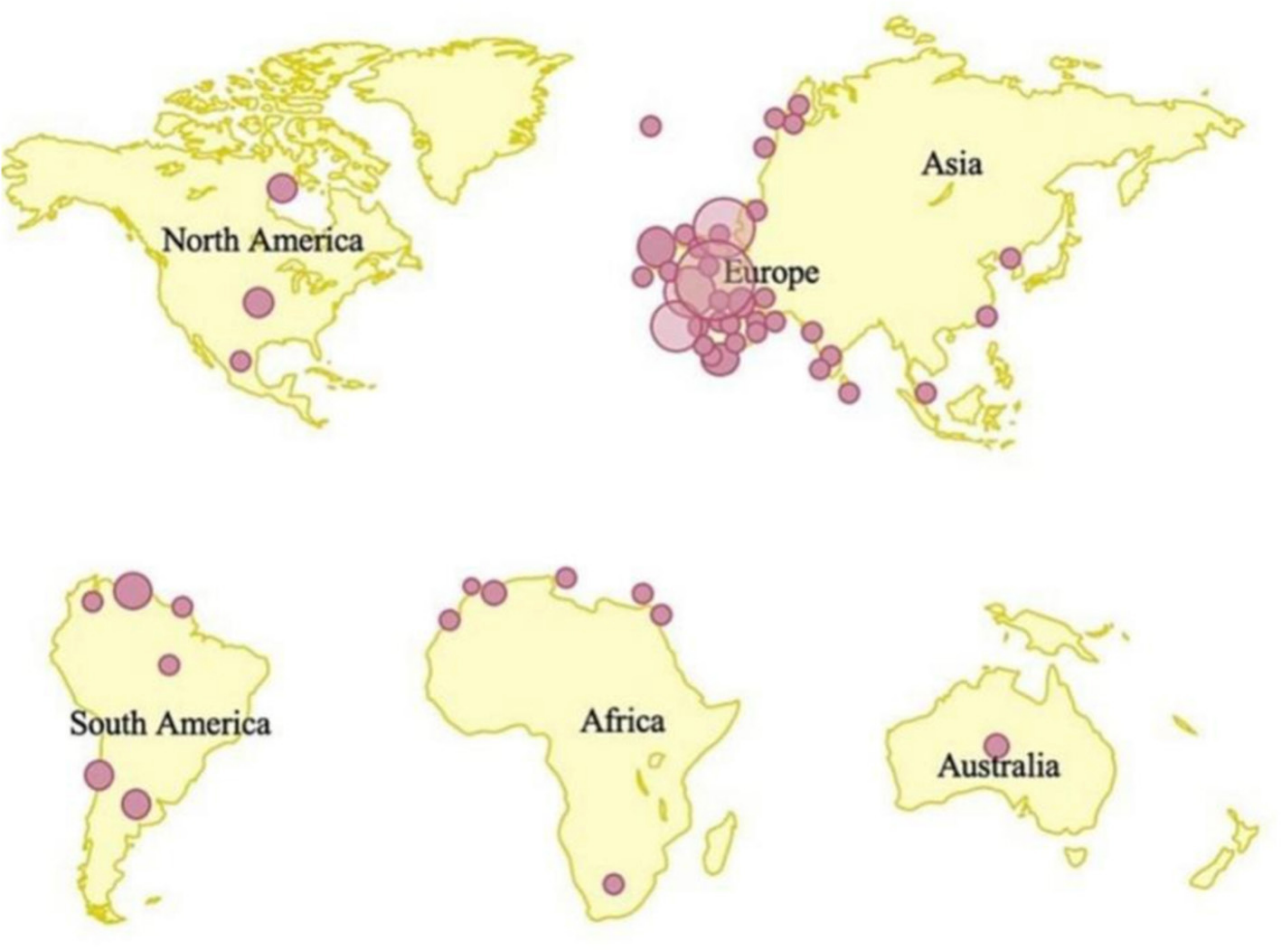
The present regionalized spatial distribution of MPX-confirmed cases.

Another issue that may cause the disease to re-emerge is a failure to offer vaccination to susceptible persons in places where human immunodeficiency virus (HIV) infection is widespread. Insufficient studies have been devoted to producing a specialized vaccine to prevent the infection (Heymann et al., 1998), given the recent return of infectious illnesses during an epidemic. Moreover, routine vaccination is currently not available in endemic countries having limited healthcare resources (Damon, 2011). The extent of protection against the MPXV outbreak offered by vaccines remains unclear. Similarly, there is currently no specific treatment approved for MPXV infection, though there are several antivirals that have been developed and are being tested to treat smallpox, including tecovirimat, brincidofovir, and cidofovir (Adler et al., 2022). The present regionalized spatial distribution of MPX-confirmed cases is shown in Figure 1 (Kaler et al., 2022).

## 3. Epidemiological dynamics of MPX

Monkeypox (MPX) is an infection of global public health significance as it not only affects states in central and western African regions, but also the contemporary globe is under threat (Reynolds et al., 2007). In the previous era, the frequency of human monkeypox (HMPX) infection was sporadic, and irregular cases were investigated in several African states. The first HMPX case was recognized in the 1970s in the Democratic Republic of the Congo (DRC), associated with a 9-month-old child (Foster et al., 1972; Arita et al., 1985). This investigation was expanded to further irregular cases recorded in 11 other states of Africa including Gabon, Cameroon, Benin, the Central African Republic, DRC, the Republic of the Congo, South Sudan, Nigeria, Côte d'Ivoire, Sierra Leone, and Liberia (Durski et al., 2018; World Health Organization, 2022a). From February 1996 to February 1997, a huge outbreak of MPX was recorded in the DRC, and ~511 infected cases were investigated (Centers for Disease Control Prevention (CDC), 2022c). A systemic review and meta-analysis (Sham et al., 2022) explained details of suspected, confirmed, and fatal MPX cases by country and year-wise.

In 2003, an MPX epidemic occurred in the US, with 47 apparent or confirmed cases. Investigation showed that the affected individuals were exposed to the virus via pet prairie dogs retained with other mammals in a pet supply capacity, comprising the primary host and rodents from African Ghana (Bernard and Anderson, 2006). Petersen et al. (2019) investigated 116 clinically verified individuals with a death rate of 6.7%, and ~280 suspicious cases appeared in Nigerian territory in 2018, with the large majority of cases in individuals under 40 years of age.

The frequency of the infection has affectedly amplified, and the DRC recorded 20 times more cases between 1981 and 1986 (7.2 cases per 100,000 people) and 2006 and 2007 (144.2 cases per 100,000 people), and a 5-fold rise from 2001 (0.64 cases per 100,000 people) to 2012 (3.11 cases per100, 000 people) (Hoff et al., 2017). Bunge et al. (2022) collected data from 28 available manuscripts and 15 gray database studies on HMPX infection point out that the incidence rate has been amplified since 1970s, with a rise in the intermediate age of infected individuals from 4 years old in the 1970s to 21 years old from 2010 to 2019. Table 1 adopted from Brown and Leggat (2016); Beer and Rao (2019); Adegboye et al. (2022) and Hatmal et al. (2022) shows the incidence of MPX and the number of deaths from 1970 to 2021.

**Table 1:** Incidence of MPX and the number of deaths from 1970 to 2021.

**Country/Region**	**Timeframe**	**Total cases**	**Total deaths**	**References**
Democratic Republic of the Congo (DRC)	1970	1	1	(Ladnyj et al., 1972)
	1981–1986	338	33	(Jezek and Fenner, 1988)
	1996–1997	773	8	(CDC, 1997)
	2001–2013	19,646	335	(Hoff et al., 2017)
	2016	155	11	(Laudisoit et al., 2016)
	2019–2020	8388	244	(WHO, 2022g)
Central African Republic	2001	8	2	(Berthet et al., 2011; Nakoune et al., 2017)
	2010	2	0	(Berthet et al., 2011)
	2015	3	1	(Nakoune et al., 2017)
	2015–2016	62	5	(Kalthan et al., 2018; WHO, 2022h)
	2017–2018	41	1	(Durski et al., 2018; WHO, 2022i)
Republic of the Congo	2003	12	1	(Learned et al., 2005)
	2010	11	1	(Reynolds et al., 2013)
	2017	88	6	(Durski et al., 2018)
Sudan Cameroon	2005	37	0	(Formenty et al., 2010)
	1989	1	0	(Tchokoteu et al., 1991)
Gabon	1987	1	1	(Müller et al., 1988)
	1991	9	0	(Durski et al., 2018)
Nigeria	1971–1978	3	0	(Breman et al., 1980)
	2017–2018	228	6	(Alakunle et al., 2020)
Sierra Leone	1970–1971	1	0	(Breman et al., 1980)
	2014–2017	2	1	(Durski et al., 2018)
Liberia	1970–1971	4	0	(Breman et al., 1980)
Côte d'Ivoire	1971	1	0	(Breman et al., 1977)
USA	2003	47	0	(Reed et al., 2004; Sejvar et al., 2004)
	2021	2	0	(World Health Organization, 2022e)
Singapore	2019	1	0	(Yong et al., 2020)
UK	2018	4	0	(Vaughan et al., 2020)
	2019	1	0	(UK, 2022)
	2021	3	0	(Yong et al., 2020)

Source: Adopted from Brown and Leggat (2016), Beer and Rao (2019), Adegboye et al. (2022), and Hatmal et al. (2022).

In the retrospective outbreak investigations, MPX was recorded in youngsters and teenagers in the enzootic areas, with the same clinical picture and symptoms as observed in older individuals. The WHO has lately documented that serious cases of MPX more frequently occur among youngsters and are associated with the level of virus contact. Moreover, the severity of MPX cases may be associated with individual health conditions, the nature of complexities, and essential immune insufficiencies (WHO, 2022c). Individuals whose date of birth was after the 1980s are at greater risk because immunization for SPX stopped after its eradication, and this immunization can also defend people against MPX (Simpson et al., 2020). Additionally, it was thought that MPX infects women and men similarly, but, in the recent multi-country epidemic, several MPX cases have been reported in men who have sex with other men (MSM) (Bunge et al., 2022; Perez Duque et al., 2022; WHO, 2022c; Xiang and White, 2022). As per the CDC report on the 2022 outbreak, the majority of MPX cases are due to MSM, which puts bisexual, transgender, and gay individuals at a greater threat of MPX (CDC, 2022b). Further investigations are mandatory for a better understanding of risk factors regarding sexual transmission dynamics of MPXV among MSM. The multi-state 2022 epidemic of MPX cases and deaths recorded by WHO (2022e) is shows in Table 2.

**Table 2:** MPX cases and deaths recorded by the WHO during the multi-state 2022 epidemic (as of 8 June 2022) (World Health Organization, 2022e).

**WHO zone**	**State**	**Confirmed cases**	**Suspected cases**	**Deaths**
AFRO	Liberia	0	4	0
	Sierra Leone	0	2	0
	Republic of Congo	2	7	3
	DRC	10	1,356	64
	Central African Republic	8	17	2
	Ghana	5	12	0
	Nigeria	31	110	1
	Cameroon	3	28	2
AMRO	Argentina	2	0	0
	Canada	110	0	0
	Mexico	1	0	0
	USA	40	0	0
EMRO	UAE	13	0	0
	Morocco	1	0	0
EURO	Austria	1	0	0
	Belgium	24	0	0
	Czech Republic	6	0	0
	Denmark	3	0	0
	Finland	3	0	0
	France	66	0	0
	Germany	113	0	0
	Hungary	2	0	0
	Ireland	9	0	0
	Italy	29	0	0
	Israel	2	0	0
	Latvia	2	0	0
	Malta	1	0	0
	Netherlands	54	0	0
	Norway	2	0	0
	Portugal	191	0	0
	Slovenia	6	0	0
	Spain	259	0	0
	Sweden	6	0	0
	Switzerland	12	0	0
	UK	321	0	0
WPRO	Australia	6	1	0
Cumulative	36 countries	1,344	1,537	72

In some investigations, there is evidence of mixed infection of MPX with other blood-borne diseases and some sexually transmitted diseases (Liu et al., 2022), and people with HIV infection reflected a greater risk dynamic for MPX in the recent epidemic (Khaity et al., 2022; Bragazzi et al., 2023). In advanced cases of uncontrolled HIV infection, inappropriate immune response is significantly related to a weak prognosis, a longer period of disease signs, late curing of self-controlling MPX, and complex cures (Iñigo Martínez et al., 2022; Liu et al., 2022). Consequently, sorting MPX cases for HIV is extremely suggested in MSM (Liu et al., 2022). Recently, MPX has been accepted as a key factor that escalates the chance of contracting HIV (Davido et al., 2022; Patrocinio-Jesus and Peruzzu, 2022). A recent epidemiological study from Madrid, Spain reported that 44.3% (225 cases out of 508 totals) of MPX-confirmed cases were linked to HIV infection (Iñigo Martínez et al., 2022). An additional report from London, UK indicated that 35.9% (70 cases out of 195 totals) of MPX-confirmed cases were linked to HIV infection (Patel et al., 2022). Similarly, mild infections of MPX among individuals with HIV and AIDS have been documented in Italy and Portugal (Antinori et al., 2022; Perez Duque et al., 2022), particularly among people with enhanced T-helper cell count, untraceable HIV viral genomic substance, and weak anti-retroviral treatment (Ortiz-Martínez et al., 2022). Infected individuals with immunological suppression initiated by HIV presented a clear-cut, wide scale of clinical appearances and characteristic MPX wounds. Fever, exanthema, inguinal lymphadenopathy, and genital ulcers were major clinical appearances in MPX-infected individuals during the epidemic in Portugal (Perez Duque et al., 2022). Pustules, papules, and a necrotic centralized wound in the perianal region, trunk, genitals, mouth, and facial region were recorded in a 24 year old bisexual man with HIV infection (De Sousa et al., 2022). Moreover, throughout the 2017–2018 MPX outbreaks in Nigerian regions, the majority of mortalities related to MPX were in individuals with unrestrained HIV, with AIDS appearances, who were not receiving proper medication (Yinka-Ogunleye et al., 2019). Another study on Nigeria showed that mixed HIV-infected MPX cases had a more prolonged disorder, greater wounds, and greater frequency of both genital ulcers and bacterial skin diseases, compared with HIV-negative MPX-infected individuals (Ogoina et al., 2020). Mixed infection with other sexually transmitted diseases (STDs) was also documented among MPX and HIV cases. An infected individual with unidentified progressive HIV and syphilis presented with a severed penis, oral mucosal infection, nasal necrotic wound, and MPX lesions spread over the entire body (Boesecke et al., 2022).

Active surveillance of MPX was carried out in nine regions of central DRC during 2005–2007, and ~760 MPX confirmed cases were recorded, with an annual occurrence of 55.3 per 100,000 people. Male gender, age <15, a history of vaccination against SPX, and inhabitants of afforested regions were the main associated risk factors of MPX (Rimoin et al., 2010). In 2017, a huge incidence of MPX was recorded in the Nigerian regions, with over 500 suspicious, over 200 confirmed cases, and a death rate of 3% (World Health Organization, 2022b). In an additional study, Beer and Rao (2019) investigated 71 reports relating to MPX cases and local epidemics during 1970–2018. The rates of documented occurrences were amplified since 1970, with an overall of 35 recorded epidemics outside the DRC, with 20 between 2010 and 2018.

The CDC, from 1 January 2022 to 5 August 2022, documented 28,220 confirmed cases of MPX in 88 states of the world (CDC, 2022e). The majority of these cases (27,875) were documented in 81 states that have not retrospectively documented MPX (CDC, 2022e). Additionally, a few months ago, the WHO investigated various human MPX outbreaks in different regions of Europe, the Americas, the Eastern Mediterranean, and the Western Pacific, with a total of 1,285 MPX confirmed cases, while 59 confirmed and 1,536 suspicious MPX cases were recorded, with 72 deaths occurring in African territories from January 2022 to June 2022 (World Health Organization, 2022e). The host range and susceptibility to MPXV infection was detected during investigatory research in the laboratory by Silva et al. (2020) shown in Table 3. Several eco-bionomical, environmental, and geostrategic dynamics might have led to the regional and global appearance and re-appearance of MPX infection, including the misuse of rain timberlands, climate alteration, civil and military clashes in disease areas, highly mobile populations, declining herd immunity, and the ceasing of SPX immunization (Fauci, 2005; Liu et al., 2022). On the contrary, the reservoir host, natural history, and pathogenesis of MPXV are uncertain; hence, there are significant disputes in recognizing the epidemiological dynamics of MPX infection (Petersen et al., 2019).

**Table 3:** Host range and animals susceptible to MPXV infection (Silva et al., 2020).

**Order/Family**	**Species**	Tool of investigation^*^	Relationship to human infection^**^
Hominidae/Primates	*Homo sapiens* (Humans)	Virus isolation	Yes
	*Pongo pygmaeus* (Orangutans)	Virus isolation	Yes
	*Pan troglodytes* (Chimpanzees)	Virus isolation	No
Cercopithecidae/Primates	*Cercocebus atys* (Sooty mangabeys)	PCR/virus isolation	No
	*Macaca fascicularis* (Cynomolgus monkeys)	Virus isolation	Yes
Callithrichidae/Primates	*Callithrix jacchus* (White-tufted marmosets)	Lab. infection	No
Chinchillidae/Rodentia	*Oryctolagus cuniculus* (Rabbits)	Lab. infection	No
Muridae/Rodentia	*Mus musculus* (Inbred mouses)	Lab. infection	No
Cricetidae/Rodentia	Hamsters	Lab. infection	No
Nesomyidae/Rodentia	*Cricetomys sp*. (Giant-pouched rats)	PCR/virus isolation	No
Gliridae/Rodentia	*Graphiurus sp*. (African dormices)	PCR/virus isolation	No
Sciuridae/Rodentia	*Funisciurus sp*. (Rope squirrels)	PCR/virus isolation	Yes
	*Cynomys ludovicianus* (Black-tailed prairie dogs)	PCR	Yes
	*Marmota monax* (Woodchucks)	PCR/ virus isolation	No
Dipodidae/Rodentia	*Jaculus sp*. (Jerboas)	PCR/ virus isolation	No
Hystricidae/Rodentia	*Atherurus africanus* (Porcupines)	PCR/virus isolation	No
Macroscelididae/Pilosa	*Myrmecophaga tridactyla* (Ant-eaters)	Virus isolation	No
Didelphidae/Didelphimorphia	*Didelphis marsupialis* (Southern opossums)	PCR/ virus isolation	No
	*Monodelphis domestica* (Shot-tailed opossums)	PCR/virus isolation	No
Erinaceidae/Erinaceomorpha	*Atelerix sp*. (African hedgehogs)	PCR/virus isolation	No

* Tool of investigation: virus isolation from naturally infected animals; laboratory infection; or molecular assay (PCR). Susceptibility to MPXV infection was detected during investigational research in the laboratory.

** Transmission to humans previously described in the literature (Silva et al., 2020).

### 3.1. Epidemiological dynamics of MPX retrospective to the global epidemic in 2022

Based on 50 years of retrospective analysis of MPX, the DRC has been the single state to constantly investigate HMPX patients, and, in the previous 30 years, the figure for documented infected individuals was over 1,000 per annum (Bunge et al., 2022; WHO, 2022f). During the year 2020, ~6,257 suspicious individuals of HMPX were investigated in the DRC (WHO, 2022f). In the initial 120 days of 2022, ~1,238 Central African clade-associated new MPX cases were documented in the DRC (Bunge et al., 2022; World Health Organization, 2022a).

Human monkeypox (HMPX) was only reported outside the African region when outbreaks linked to infected pet prairie dogs increased in the USA in 2003 (Brown and Leggat, 2016; Centers for Disease Control Prevention (CDC), 2020). None of the cases in this outbreak (a total of 81 recognized cases, 40% of which were confirmed cases) were attributed to secondary transmission, and the mortality rate was zero. The dogs acquired infections from infected exotic dormice and pouched rats, which were transported from Ghana.

Multiple factors are involved in the rise of HMPX since the 1970s. These include active, passive, and sentinel surveillance efforts, climatic dynamics, deforestation, and rapid demographic expansion of regions where the MPXV is retained in a huge population of host animals, with a surge in natural or incidental hosts. Furthermore, individuals aged 40–45 years or less lack immunity to the smallpox virus after the termination of immunization against smallpox in the 1980s. In summary, significantly associated dynamics also involve hominid behavior (for example, interaction with dead or live creatures, reservoir hosts, staying in tropically reforested or newly desforested ranges, hunting, close interaction with an infected individual, sharing a joint bedroom with an infected individual, sharing kitchenette kits with an infected individual, and preparation and intake of bush meat or monkeys), scarcity, military, and political conflicts, territorial movements, tourism, the trade of exotic animals, and public healthcare services (Hutin et al., 2001; Parker et al., 2007; Rimoin et al., 2010; Vaughan et al., 2020; Mauldin et al., 2022; Quarleri et al., 2022).

### 3.2. Epidemiological dynamics of MPX in the global epidemic in 2022

Since May 2022, many outbreaks of HMPX have been documented in European states for the first time, where the MPX infection is not prevalent (ECDC, 2022a; Sham et al., 2022; World Health Organization, 2022b,j). From 13 May 2022 to 16 May 2022, the UK documented six HMPX cases for the first time; these cases were investigated without any epidemiological associations with imported animals, travel to African countries, and with all cases self-distinguishing as men who have sex with other men, bisexual, or gay (WHO, 2022c). The majority of HMPX cases have a travel record to various states in Europe and America. Moreover, cases of HMPX in the enzootic world remain to be described.

Since early May and as of 19 September 2022, over 62,000 HMPX cases have been documented in the non-endemic world (Centers for Disease Control and Prevention (CDC), 2022d). As of 19 September 2022, ~44 European republics have documented 24,017 cases, demonstrating 38.5% of all the globally documented cases in the recent epidemic. The highest figure (n = 6947) was documented in Spain, followed by France (n = 3898), Germany (n = 3563), and the UK (n = 3552); however, one case each was documented in Ukraine and Turkey. In this epidemic, the largest number of cases (n = 23,892) was documented in the USA, comprising 38.3% of the globally reported MPX cases. Variations in the incidence rate of HMPX by state might be relatively described by dissimilarities in demography and density population at threat, social and economic circumstances, under-diagnosis, and/or improper reporting.

The person-to-person transmission dynamic of HMPX has been documented in the European region for the first time (ECDC, 2022a; Vivancos et al., 2022). In the recent occurrence, clinical features that differ from retrospective documentations were investigated, including the lack of prodromal or very minor prodromic symptoms, a rash that appears earlier than the prodromic stage, a rash that exhibits only an ulcer or some abrasions, a skin rash restricted only to the perineal or anogenital region, and mainly inguinal site lymphadenopathy (Bunge et al., 2022; Iñigo Martínez et al., 2022; Thornhill et al., 2022). Based on the severity, MPX is categorized as mild and moderate, with ~4 to 10% of patients admitted to hospitals (Centers for Disease Control and Prevention (CDC), 2022d; Girometti et al., 2022; WHO, 2022d). Due to encephalitis and comorbidities, ~20 deaths due to MPX have been documented in the current multiregional epidemic, a figure that matches that in Africa as well as in non-endemic states (Centers for Disease Control and Prevention (CDC), 2022d; ECDC, 2022a; European Centre for Disease Prevention Control (ECDC), 2022c). Though several documentations specified a small number of cases without symptoms (Centers for Disease Control and Prevention (CDC), 2022d), a UK-based cohort study investigation showed interactions with an individual with confirmed MPX infection were recorded in ~25% of cases (Patel et al., 2022). In this prospective epidemic, there has been no concrete evidence of animal-to-human or human-to-animal spread. In this occurrence, the investigated viruses were linked to the West African clade (Isidro et al., 2022; Kmiec and Kirchhoff, 2022).

An epidemiological study at 43 locations in 16 investigated states documented that ~99% of men were affected by MPX, among whom 98% self-distinguished as bisexual men or gay, or men who have sex with other men (Thornhill et al., 2022). In the current study, the 18–50 years of age range was reported as having an average of 38 years of age. Among them, 41% closely interacted with HIV patients, and in most of the cases, HIV was considerably controlled. Pre-exposure prevention protocol was adopted by 57% of HIV-negative individuals or those patients who were not aware of their HIV status. In 29% of examined individuals, there was evidence of associated sexually communicated infections. In this study, confirmation of sexual transmission of infection was impossible, sex-related history was investigated in 95% of5 patients, 20% reported engaging in “chem sex” (sex-linked with the practice of medicines), and 32% reported attending on-site-sex events (Thornhill et al., 2022).

In the Spanish outbreak (Iñigo Martínez et al., 2022), ~84.1% of MPX cases were documented as having a history of condomless sex or having sex with more than one sex partner within 3 weeks before the beginning of disease indications, 8.1% of infected persons confirmed having safe sexual activities, and 7.9% gave no response. Furthermore, in the present report, ~80.3% of individuals were not aware of MPX or had no interaction with a recognized MPX case. One month before MPX diagnosis, numerous individuals had an international travel history to Italy, the UK, Germany, Belgium, Portugal, Peru, etc., with no recorded cases of travel to African countries. Furthermore, at a sauna region in Madrid and at the Gay Pride festival on a Spanish island, some cases of MPX were reported, with various secret gatherings also having a major role; dating via social networks was recorded by 56.9% of individuals as well as sexual activities in bars, touring zones, and secret studios. In this occurrence, the MPXV was investigated in seminal fluid samples of the patients, with sexual interaction acts a significantly associated factor in the disease occurrence. More investigation is required to explain the sexual transmission dynamics of MPX via genital fluids (Antinori et al., 2022; Iñigo Martínez et al., 2022; Thornhill et al., 2022; Noe et al., 2023).

Remarkably, various reports show that data were registered as having a lack of immunization status (Benites-Zapata et al., 2022). Among the US MPX cases for whom immunization status was accessible, 14% testified retrospectively to being vaccinated against smallpox (with 23% receiving single instead of double doses, 23% receiving pre-exposure prevention at an unidentified stage before the current occurrence, and 54% of individuals not providing an answer about vaccination status) (Philpott et al., 2022). To date, 344 MPX cases have been recorded among medical staff, and among them, some cases of spread via job-related exposure have been described in this occurrence (Centers for Disease Control and Prevention (CDC), 2022d). Worldwide, youngsters are most vulnerable to MPX because of the termination of smallpox immunization after the eradication of smallpox (Factsheet for health professionals on monkeypox: European Centre for Disease, 2022d). To avoid MPX, two vaccines (JYNNEOS and ACAM2000) are applied as follows: JYNNEOS vaccine is applied to safeguard against both smallpox and MPX, whereas the ACAM2000 vaccine is applied to protect against smallpox (Centers for Disease Control Prevention (CDC), 2022c; Factsheet for health professionals on monkeypox: European Centre for Disease, 2022d). The feedback of the immune system after vaccination is mainly based on cross-defense among the orthopoxviruses and vaccinia virus (McCollum and Damon, 2014; ECDC, 2022a). In the ongoing occurrence of MPX in the USA, men who have sex with other men, gender-diverse individuals, or transgender individuals who had sex with men in the previous 14 days might get the vaccination if they had sex with numerous individuals, or had sex at commercial sex clubs or bathhouses, or had sexual activities at an occasion, site, or in a zone where MPX spread is happening (Centers for Disease Control Prevention (CDC), 2022c). As per the recommendations of the WHO, several states in Europe, including the UK, Germany, France, and Spain, were providing immunization during the 2022 MPX epidemic (ECDC, 2022b; Factsheet for health professionals on monkeypox: European Centre for Disease, 2022d).

The WHO measures the MPX threat as sensible worldwide, with the exemption of the European and American regions, where the threat is evaluated as high (Factsheet for health professionals on monkeypox: European Centre for Disease, 2022d; Zachary and Shenoy, 2022). The recent global occurrence differs from previous epidemics in a few ways: the infrequent degree of incidence; unusual rapid expansion globally; spreading in non-endemic countries; mostly spreading among younger men (aged 18–44 years), with over 97% of them self-recognizing as men who have sexual intercourse with other men or unsafe sex with several individuals; the role of different super spreading occasions associated with transnational get-togethers; while asymptomatic infections and lack of or mild signs throughout the prodromal period make easier the transmission dynamics of the virus; and the occurrence of minor cases (Bunge et al., 2022; Centers for Disease Control and Prevention (CDC), 2022d; Delaney et al., 2022; WHO, 2022d). In summary, an advanced investigation is required to properly recognize and advance the supervision of HMPX.

## 4. MPX host range

Monkeypox virus (MPXV) isolates based on phenotypic and genetic deviations are divided into two different clades, specifically the Congo Basin and the West African clades (Likos et al., 2005). In contrast to the variola virus, which affects only humans, the MPXV is among those orthopoxviruses that can infect numerous animal hosts and can spread to humans (Parker et al., 2007; Parker and Buller, 2013; Patrono et al., 2020; Kmiec and Kirchhoff, 2022). The fixed reservoir host of the MPXV can even be unrecognized, but some small mammalians such as giant pouched rats (*Cricetomys spp*.), rope squirrels (*Funisciurus spp*.), sun squirrels (*Heliosciurus spp*.), and African dormice (*Graphiurus spp*.) are assumed to transmit the virus to human beings in Central and West Africa (Alakunle et al., 2020). MPXV is communicated from animals to human beings during hunting, trapping, treating infected animals, and dealing with their secretory and excretory fluids.

Based on experimental analyses and field investigations, MPXV has been documented in a wide range of rodents, including *Oryctolagus cuniculus* (rabbits), *Mus musculus* (mice), *Marmota monax* (woodchucks), hamsters, *Jaculus* sp. (jerboas), and *Atherurus africanus* (porcupines). Similarly, based on techniques such as molecular assay, virus separation, or *in vitr*o contamination, vulnerability to MPXV was investigated in black-tailed prairie dogs (*Cynomys ludovicianus*), anteaters, short-tailed opossums (*Monodelphis domestica*), giant anteater (*Myrmecophaga tridactyla*), African hedgehogs (*Atelerix* sp.), southern opossums (*Didelphis marsupialis*), and various non-human primate species (Parker et al., 2007; Doty et al., 2017). The host range and susceptibility to MPXV infection is also shown in Table 3 (Silva et al., 2020).

In Africa, Asia, and Europe, non-human primates, chimpanzees (*Pan troglodytes*), orangutans (*Pongo pygmaeus*), cynomolgus monkeys (*Macaca fascicularis*), and sooty mangabeys (*Cercocebus atys*) can be infected with MPXV. In the USA and the UK, non-human primates (Magnus et al., 1959; Wachtman and Mansfield, 2012; Alakunle et al., 2020) and common marmosets (*Callithrix jacchus*) were determined to be vulnerable to MPXV by intravenous injection (Mucker et al., 2015). Non-human primates may be affected by MPXV and show signs and symptoms, while small mammalians can be asymptomatic carriers of the virus (CDC, 2022a).

In 2003, HMPX infection in the USA was mainly linked with close interaction with ill pet prairie dogs introduced from the Ghana region of West Africa (Reed et al., 2004). This incident, as well as the rodent's infection, intensified alarms about the entry of MPX infection into the USA. In the meantime, the vulnerability of numerous African rodents to MPXV raised fear related to the spread of the virus to human beings, as these rodents are often maintained as pets (Centers for Disease Control Prevention (CDC), 2020; Sklenovská, 2020). Non-human primates, squirrels, and rodents have been observed to have MPXV based on sero-investigations in African territories. Wild animals are more susceptible to the disease. In 1985, MPXV was isolated from Thomas's rope squirrel (*Funisciurus anerythrus*) in the DRC and in 2012 from the sooty mangabey (*Cercocebus atys*) in Cote d Ivoire, signifying that these animal species might act as MPXV reservoirs hosts (Falendysz et al., 2017).

Human beings can also be accidental hosts (Parker et al., 2007) since the eradication of smallpox, based on MPXV morbidity and mortality, it is converted into the most significant infective zoonotic orthopoxvirus for humans. In 1970, the first human case of MPXV was documented in a 9-month-old child in the DRC, who presented with smallpox-like skin lesions (Arita and Henderson, 1968; Ladnyj et al., 1972). Numerous humanoid cases were investigated in subsequent years. During 1970–1999, the WHO documented almost 404 confirmed and 500 suspicious cases of human MPXV in various African states (Liberia, Gabon, Côte d'Ivoire, Central African Republic, and Cameroon, but predominantly in the DRC) (World Health Organization, 1997; Heymann et al., 1998; Sklenovská and Van Ranst, 2018). In May and June 2003, some MPX cases were reported to the Wisconsin Division of Public Health, with no mortality and no person-to-person spread observed (Centers for Disease Control Prevention (CDC), 2022c). The origin of this occurrence was traced back to the importation of exotic infected animals from Ghana (Khodakevich et al., 1986; Sklenovská and Van Ranst, 2018; CDC, 2022b). Luckily, the stage-wise episode of infected rodents in cages in the USA was temporary, and the pattern of spread in the country was destroyed (Petersen et al., 2019a). More recently, on 27 September 2022, 66,000 cases of MPX were confirmed in more than 100 non-endemic states, with fluctuating epidemiological footprinting from retrospective outbreaks (Li et al., 2022).

Human MPX cases have been snowballing globally with time, although they might have been miscalculated. Remarkably, diagnostic capacities in the affected states are mostly inadequate, while global healthcare personnel are mostly unaware of MPX disease. The emergence of the current MPX spread is linked with dynamics such as the growing invasion of hominids into wild habitations, the international and global travel of the public from enzootic regions to non-endemic areas, the introduction of pets and laboratory animals, lack of active disease surveillance, and improper prevention and control strategies (Essbauer et al., 2010). Furthermore, the termination of smallpox immunization and various reports of animals in captivity or experimental laboratories have made the global public susceptible to MPXV infection or other orthopoxvirus infection. As the MPX virus is an increasing global zoonotic threat with epidemic potential, and as most of its host range and life cycle in nature remains unclear, developments are immediately mandatory to recognize its biological cycle and host range for future prevention and control strategy.

## 5. Associated risk factors of MPX

Although the main associated risk factors fluctuate among different epidemics, the significance of obtaining the characteristics of particular individuals for calculating and predicting epidemic patterns cannot be ignored. Conventionally, MPX cases involving spread among human beings are more probable to be individuals who are women, non-vaccinated against smallpox, living in the same house, or providing cure to a primary case (JeŽek et al., 1988). Prominently, this information is based on clade 1-associated MPX in the DRC and did not represent other enzootic regions; outbreak investigations of different endemic states show that youngsters face the ample burden of the MPX infection. In an occurrence of clade 2B-associated MPX in Nigerian territory, mostly 21 to 40-year-old individuals were involved (Alakunle et al., 2020), although the index case was an 11-year-old teenager (Ogoina et al., 2019; Hobson et al., 2021). These associated risk factors specify the role of social and behavioral determining factors in helping the person-to-person spread of MPX infection. However, a systemic review and meta-analyses (Sham et al., 2022) explained the detailed associated risk factors for the primary introduction of MPX.

One of the serious associated risk factors for patients and healthcare workers is nosocomial MPXV infections (nosocomial infections, also referred to as healthcare-associated infections (HAI), are infection(s) acquired during the process of receiving healthcare that were not present at the time of admission) in both enzootic and non-enzootic areas. Smallpox was also mainly due to nosocomial occurrences (CDC, 1963), with the peak rate of spread occurring inside health centers (Kiang, 2003). Similarly, hospital-borne occurrences of MPX are mainly serious and long-term. These consistent multifactorial results include individuals who are susceptible to diseases, healthcare center sanitation patterns, and the usage of aerosol-producing measures (Judson, 2019). A total of six generations of MPXV spread were investigated in a public healthcare center in Impfondo, Republic of Congo, specifying MPXV's potential to spread if not rapidly handled in healthcare settings (Learned et al., 2005). On one occasion in the UK, a medical employee who had collected a blanket and dressing of an MPX-infected person was subsequently contracted MPXV (Vaughan et al., 2020).

Zoonotic transmission (transmission from animals to human beings) can arise from direct interaction with the blood, body fluids, or mucosal or cutaneous lesions of infected animals (Nigeria Centre for Disease (NCDC), 2022). In Africa, MPX has been reported in various hosts including tree squirrels, rope squirrels, dormice, Gambian poached rats, several types of monkeys, and other animals (Kile et al., 2005; Yinka-Ogunleye et al., 2019). The reservoir of MPX has not cleared yet; however, rodents are the main expected but not clear yet (Kile et al., 2005). The intake of uncooked meat and other foodstuffs of infected animal origin is a probable risk factor (Petersen et al., 2018). Individuals living in or near forested regions could have an incidental or low-degree of exposure to infected animals.

Human-to-human spread can result from close contact with respiratory discharges, dermal abrasions of an infected individual, or a newly infected entity (Nolen et al., 2015). MPXV spread via respiratory particles typically requires lengthy and close interactions, which put community health staff, families, and other close contacts of active cases at greater risk (Petersen et al., 2018). The predictable sequence of spread in the public has grown in the current era from six to nine repeated human-to-human contaminations, and this could indicate decreasing protection in humans due to the end of smallpox immunization (Meslin et al., 2000).

Human-to-animal transmission of MPXV has not been reported yet and it is believed that the outbreak may not have been caused by infection from animals (Diaz-Cánova et al., 2022). European health administrators firmly recommend that rodent pets, e.g., guinea pigs and hamsters, that belong to patients with HMPX should be quarantined and watched or even euthanized to stop the spread of the virus (Heskin et al., 2022). However, the most recent identifications in August 2022 were two cases of human-to-dog transmission reported in France and Brazil (Peters, 1966; Islam et al., 2023). In Paris, a pet dog (a healthy 4-year-old male Italian Greyhound) of two individuals who were suffering from MPX was also diagnosed with MPXV. The virus was found in the individuals, and the dog showed homology on DNA sequencing (Seang et al., 2022). This dog tested positive for MPXV after showing symptoms such as abdominal abscesses. Based on the sequencing results and symptoms of the two patients as well as the dog, the researchers concluded that MPXV was indeed transmitted between humans and dogs (Seang et al., 2022).

As per disease investigations, the main concern is more for youngsters and immunocompromised adults, such as persons who have HIV infection (De Sousa et al., 2022). The recent global MPXV occurrence in human beings increases the probability that the virus might have mutated genetically and that human behavior may have altered or collected. These mutations might have occurred due to decreasing smallpox immunity, diminishing COVID-19 protective policies, sexual connections, and the restart of intercontinental movements (Zhu et al., 2022). An additional factor recognized in the current topographical distribution of MPX spread is sexual interaction, in particular among men who have sex with other men (ECDC, 2022a). Table 4 shows the updated risk factors associated with MPX cases worldwide.

**Table 4:** Risk factors associated with MPX cases.

**Risk factor**	**References**
Age	In Nigeria, the age of individuals affected by MPX was < 40 years, with the absence of cross-protective resistance as they were born after the termination of the smallpox eradication campaign (Petersen et al., 2019).
Nosocomial infection	Healthcare-associated spread (Petersen et al., 2018).
Zoonotic infection	Interaction with infected prairie dogs (Kile et al., 2005) and wildlife, bites from peri-domestic animals, hunters (Meslin et al., 2000; Reynolds et al., 2007), household materials (Quiner et al., 2017; Yinka-Ogunleye et al., 2019; Guagliardo et al., 2020), and peridomestic rodents (Reynolds et al., 2010; Salzer et al., 2013).
Travelers	Immigrants to non-endemic monkeypox regions (Alakunle et al., 2020).
Human to human transmission	Inter-human transmission (Nolen et al., 2015).
Human-to-animal transmission	Human-to-dog transmission was reported in France and Brazil (Peters, 1966; Seang et al., 2022; Islam et al., 2023).
Men who have sex with men (MSM)	MPX was spread among MSM, those who have bisexual contact, and those who have sex with everyone, including male colleagues (Endo et al., 2022), young men who have sex with other men, engage in unsafe manners and actions comprising unsafe sex, HIV positivity, and retrospective records of sexually transmitted diseases (STDs), including syphilis (Bragazzi et al., 2023).

## 6. MPXV as a potential bioweapon

Monkeypox (MPX) is no longer a rare, self-limiting disease limited to endemic countries. The MPXV is a high-danger pathogen that can spread to various regions and poses a significant threat to public health. Its ever-changing epidemiology and transmission dynamics have increased the possibility of it evolving into a much deadlier pathogen that can be used as a bioweapon due to its unanticipated development in places with no known epidemiological linkages, which permits undetected transmission for a long period and raises concerns about the virus's evolution (Ferdous et al., 2023). Despite the potential of MPXV to be used as a global bioweapon, the possibility of biological warfare and bioterrorism cannot be completely ruled out due to modern molecular biological advances and the spread of the virus to various regions due to rising globalization and cross-border animal mobility. As a result of these factors, MPXV, along with the variola virus and many other poxviruses, is on the NIH's highest danger list. The CDC has categorized it as a “select agent.” Human travel is prevalent today, providing risk for the spread of MPX, and animals carried across borders represent an immediate danger of disease spread (Amir et al., 2023; Khattak et al., 2023).

## 7. Critical challenges associated with MPX research

To better understand the dynamics of MPX transmission and control, operational research is currently facing challenges, such as insufficient resources for detailed case investigations and contact follow-up in affected communities. A lack of adequate diagnostic facilities in laboratories is a serious problem. Owing to the lack of laboratory diagnosis capacity and access, as well as the difficulty of diagnosing MPX, it is difficult to discover any underlying etiology. A seroprevalence study would help to understand the epidemiology as well as subclinical infection among contacts in communities (Lederman et al., 2007). The currently available serological assays are generic orthopox tests; they do not specifically test for the MPXV. This is due to the fact that there is cross-reactivity between MPX and smallpox viruses, and therefore, we cannot distinguish between a MPXV infection and prior smallpox vaccinations or other orthopoxvirus infections. In addition, these assays are not currently available in the marketplace. It has been found that, according to data collected from Nigeria, ~20% of 70 MPX-negative patients presenting rash illness with similar antigens also had orthopox antibodies. To identify the transmission of other orthopoxviruses in human and animal populations, further research, including using molecular and genomic approaches, is needed (Ihekweazu et al., 2020).

Precautions such as avoiding close interaction with reservoir hosts and infected persons, proper handwashing and disinfection, avoiding non-important travel, usage of suitable personal protective equipment, appropriate practices of waste management, and quarantine, treatment, and immunization of infected individuals must be applied to reduce the spread of MPXV. It is necessary to enhance continuous active investigation and monitoring of the MPXV in community health services and in the general population, particularly in livestock populations such as animal farmhouses, marketplaces, and slaughterhouses. Individuals traveling from regions of the world where the infection is prevalent must be tested and declared free of disease before entry to another country. Infected persons must be supervised to stop the further spread of the virus to vulnerable populations. The public must be made aware of and educated on the threats of bushmeat intake, zoonotic spread, the significance of one's health, and the application of protective procedures and biosecurity against the MPXV. Finally, training public health facilitators on how to avoid the spread of the disease and how to protect themselves from the threat of infection is critical because they are at greater threat of being infected (Idris and Adesola, 2022).

## 8. Conclusion

The contemporary global public in the present era has already survived the COVID-19 pandemic and the extraordinary damages it produced. Due to globalization, communicable infections are becoming more widespread and pose a global public health threat. There is no method to determine subsequent emerging diseases, but one example, COVID-19, has re-taught the globe that what virus will arise as a major public health threat is somewhat unpredictable and that it is frequently too late to put in place counter-measures after the fact. The unpredicted appearance of MPX in the non-endemic world suggests some undetectable transmission dynamics. Hence, open-minded and vigilant epidemiological attention and global public awareness of the recent MPX epidemic are required, not only in developed economies but also in underdeveloped states that have been dominated by such viruses for several years. There is an urgent need for researchers and epidemiologists to participate more in this global public health threat, follow up on it, and conduct more molecular epidemiological research on the topic. Therefore, there is an urgent need for proper epidemiological approaches to be adopted to investigate the emergence of current MPX epidemics, as well as the true cause of the disease, transmission dynamics, identification of associated risk factors, and investigation of the global host range. Rapid documentation of new cases, active investigation, and syndromic observational surveillance approaches would provide insights into variations in epidemiological tendencies, particularly in situations where validating diagnostic techniques is challenging. Therefore, this review has been compiled to highlight the epidemiology, global host ranges, and associated risk factors of MPX, focusing on its epidemic potential and global public health threat.

## Author contributions

Conceptualization: MU and ZZ. Methodology, investigation, data curation, and writing-original review draft preparation: MU, YL, and ZZ. Writing-review and editing and visualization: MU and KM. Funding acquisition: ZZ and YL. All authors have read and agreed to the published version of the manuscript.
